# Data on the phylogenetic typing, integron gene cassette array analysis, multi-drug resistance analysis and correlation between antimicrobial resistance determinants in *Klebsiella* strains

**DOI:** 10.1016/j.dib.2016.07.016

**Published:** 2016-08-03

**Authors:** Hao Wu, Mingyu Wang, Yuqing Liu, Xinhua Wang, Yunkun Wang, Jinxing Lu, Hai Xu

**Affiliations:** a State Key Laboratory of Microbial Technology, School of Life Sciences, Shandong University, Jinan 250100, Shandong, PR China; b Shandong Key Laboratory of Animal Disease Control and Breeding, Institute of Animal Science and Veterinary Medicine, Shandong Academy of Agricultural Sciences, Jinan 250100, Shandong, PR China; c School of Environmental Science and Engineering, Shandong University, Jinan 250100, Shandong, PR China; d State Key Laboratory for Infectious Disease Prevention and Control, and National Institute for Communicable Disease Control and Prevention, Chinese Center for Disease Control and Prevention, Beijing 102206, PR China

**Keywords:** Antimicrobial resistant *Klebsiella* species, Polymerase chain reaction-restriction fragment length polymorphism (PCR-RFLP), Gene cassette arrays of integron, Statistical analysis

## Abstract

The antimicrobial resistance of *Klebsiella* species in the poultry industry is becoming a public concern. In support our recent publication “Characterization of antimicrobial resistance in *Klebsiella* species isolated from chicken broilers” (Wu et al., 2016) [1], multilocus sequence typing (MLST) and *gyrA* PCR-RFLP assays were conducted to identify the genetic relationships between and phylogenetic groups of the 90 antimicrobial resistant *Klebsiella* species isolated from a commercial broiler slaughter plant in Shandong, China. In addition, PCR-RFLP was performed to identify different gene cassette arrays in class 1 and 2 integrons, and the correlations between different antimicrobial resistance determinants were analyzed.

**Specifications Table**

**Table:** 

Subject area	Microbiology
More specific subject area	Food safety, antibiotic resistance
Type of data	Table, figure
How data was acquired	PCR, sequencing and statistical analysis
Data format	Analyzed
Experimental factors	Polymerase chain reaction-restriction fragment length polymorphism (PCR-RFLP), chi-square tests using SPSS
Experimental features	Identification of phylogenetic groups and different gene cassette arrays in class 1 and 2 integrons of *Klebsiella* species, analysis of the correlations between different antimicrobial resistance determinants
Data source location	Jinan, Shandong province of China.
Data accessibility	The data is available with this article

**Value of the data**

• The *gyrA* PCR-RFLP assay and MLST analysis in the *Klebsiella* isolates indicate the relationship of epidemiology of drug resistant bacteria in between clinical and poultry industry.
• The PCR-PFLP by *Eco*RII can be applied as a tool for detection of gene cassette arrays of integron 1 or 2.
• The statistical data and finding of a significant association of antimicrobial resistance determinants can be used as references for the investigation of other drug resistant bacteria.

## 1 Data

**Fig. 1:**
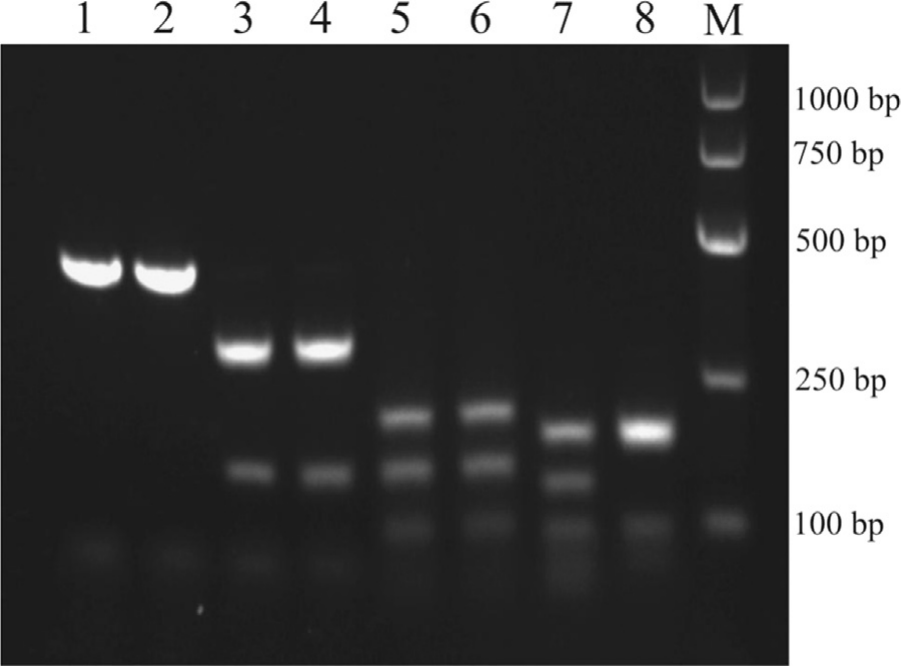
PCR-RFLP profiles of the *gyrA* gene identified in the 90 *Klebsiella* isolates using *Hinc*II, *Taq*I, and *Hae*III. Lane 1, 3, 5, 7 for KpI (89 isolates) and lanes 2, 4, 6, 8 for KpIII (one isolate). Lanes 1 and 2, the 441-bp PCR product of the *gyrA* gene. Lanes 3 and 4, *Hinc*II restriction profiles (298- and 143-bp fragments). Lanes 5 and 6, *Taq*I restriction profiles (197-, 142-, and 93-bp fragments). Lane 7, *Hae*III restriction profile (175-, 129-, 92-, and 45-bp fragments). Lane 8, *Hae*III restriction profile (175-, 174-, and 92-bp fragments). *M*, molecular size marker.

**Fig. 2:**
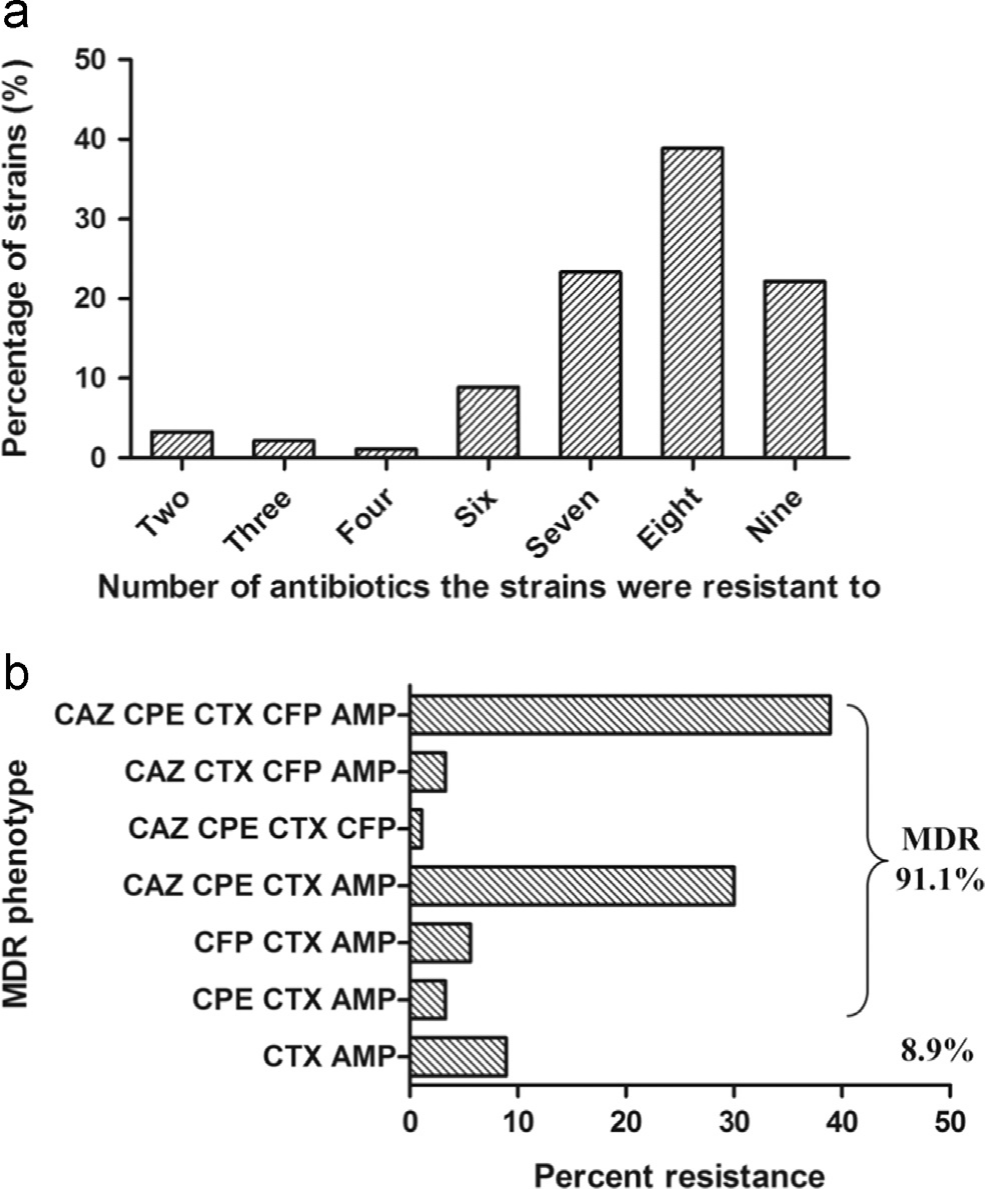
Antimicrobial resistance to different antibiotics of 90 *Klebsiella* isolates. (a) The percentage of tested strains resistant to different numbers of antibiotics. (b) The percentage of tested strains resistant to different beta-lactam antibiotic groups. CAZ, ceftazidime; CFP, cefoperazone; CTX, cefotaxime; CPE, cefepime; AMP, ampicillin; KAN, kanamycin; CHL, chloramphenicol; TET, tetracycline; CIP, ciprofloxacin.

**Fig. 3:**
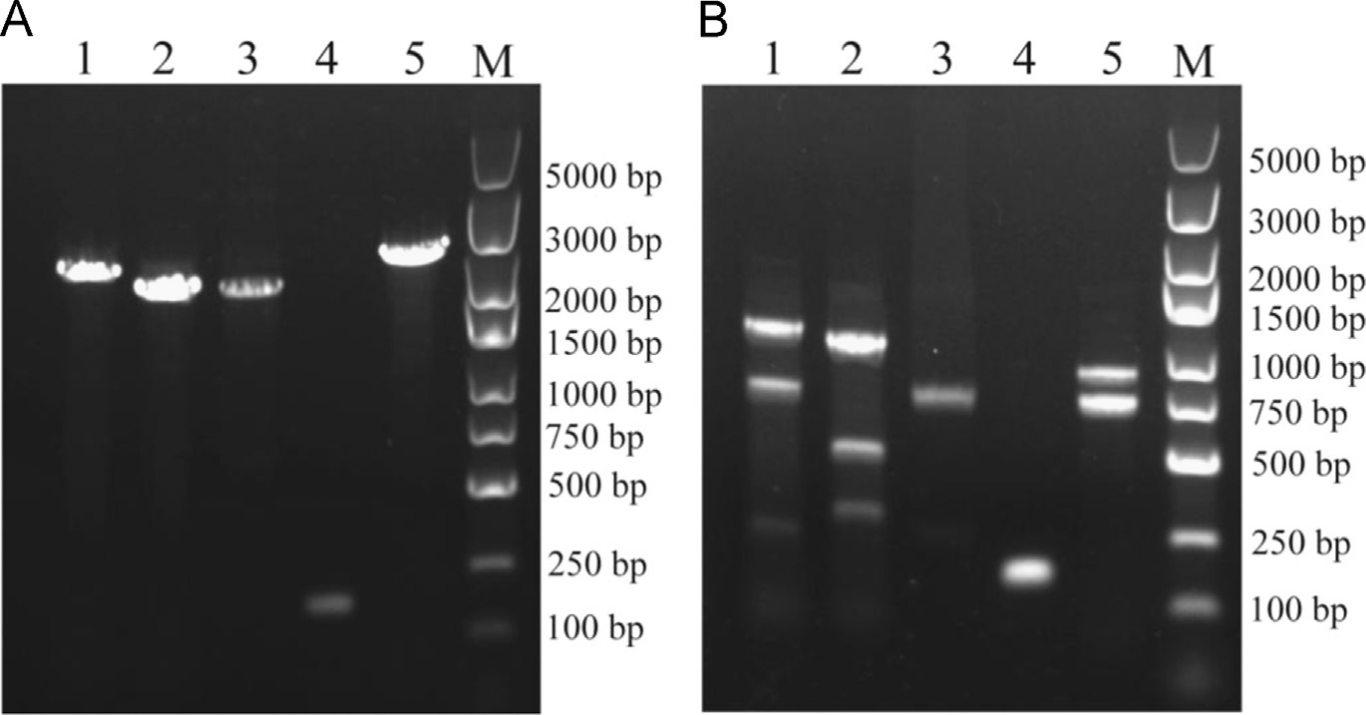
PCR-PFLP patterns of gene cassette arrays in identified integrons. Panel A, products of the PCR amplification of the variable regions of integrons. Panel B, *Eco*RII-digested restriction fragment length polymorphism patterns. Lanes 1–4 are type 1 integrons with the following gene cassettes: *dfrA17–aadA5*, *dfrA12–orfF–aadA2*, and *dfrA1–aadA1*, and empty, respectively. Lane 5 is a type 2 integron with a *dfrA1–sat2–aadA1* gene cassette. *M*, molecular size marker.

MLST was performed using seven housekeeping genes (*rpoB, gapA, mdh, pgi, phoE, infB, and tonB*), and primers of those genes for PCR amplification and sequencing were designed (Table 1) [2]. *gyrA* PCR-RFLP profiles showed nearly all (89/90) of the isolates were identified as KpI-type and only one isolate was KpIII (Fig. 1). Antimicrobial susceptibility to nine antimicrobial agents was tested for the 90 *Klebsiella* isolates [1]. Among the isolates, 96.7% of them were resistant to more than three tested antimicrobial agents as well as 91.1% were resistant to more than three beta-lactam antibiotics (Fig. 2). A significant association between different antimicrobial resistance determinants was analyzed (Table 2). PCR-PFLP patterns of gene cassette arrays for integron 1 or 2 were performed (Fig. 3), and the detailed description was in the original article [1].

## 2 Experimental design, materials and methods

### 2.1 PCR Program

PCRs were prepared as follows: a final volume of 25 μl containing 1 μM of each primer, 0.2 mM dNTPs, 1.5 mM MgCl_2_, and 1 unit of *Taq* polymerase (TransGen Biotech, Beijing, China). The conditions used for amplification were as described by the original article [1].

### 2.2 Primers designed for the MLST analysis of Klebsiella isolates

The primer pairs for seven housekeeping genes (*rpoB, gapA, mdh, pgi, phoE, infB, and tonB*) were designed for PCR amplification and sequencing (Table 1), as described previously [2].

### 2.3 Molecular identification by PCR-RFLP analysis of the gyrA gene

*gyrA* PCR-RFLP patterns were obtained by the restriction analysis of a 441-bp PCR fragment of the *gyrA* gene using the restriction enzymes *Hinc*II, *Taq*I, or *Hae*III (Fig. 1) [3], [4]. According to this approach, *Klebsiella* strains can be classified into the KpI, KpII, and KpIII phylogenetic groups.

### 2.4 Statistics analysis

According to the prevalence of antimicrobial resistance genes among 90 *Klebsiella* isolates [1], the number of antimicrobial resistance strains (Fig. 2a) and the percentage of tested strains resistant to different numbers of beta-lactam antibiotic groups were analyzed (Fig. 2b). Also the statistical analysis of the correlation between different antimicrobial resistance determinants was performed by chi-square tests using SPSS (SPSS 19.0 for Windows; SPSS Inc., Chicago, IL, USA), and a *p*-value <0.05 was considered to be statistically significant (Table 2).

### 2.5 Identification of integron gene cassette arrays

The gene cassette arrays of class 1 and 2 integrons were analyzed (Fig. 3) by a PCR-RFLP method as described previously [5].

## Figures and Tables

**Table 1:** Primers used in the MLST analysis of *Klebsiella* isolates.

Locus	Putative function of gene	Primer sequence (5′–3′)^a^		No. of alleles	Amplicon size (bp)	Melting temp (°C)
*rpoB*	Beta-subunit of RNA polymerase B	VIC3	GGCGAA ATGGCWGAGAACCA	4	501	51
		VIC2	GAGTCTTCGAAGTTGTAACC			
*gapA*	Glyceraldehyde 3-phosphate dehydrogenase	gapA173	TGAAATATGACTCCACTCACGG	5	450	60
		gapA181	CTTCAGAAGCGGCTTTGATGGCTT			
*mdh*	Malate dehydrogenase	mdh130	CCCAACTCGCTTCAGGTTCAG	4	477	50
		mdh867	CCGTTTTTCCCCAGCAGCAG			
*pgi*	Phosphoglucose isomerase	pgi1F	GAGAAAAACCTGCCTGTACTGCTGGC	5	432	50
		pgi1R	CGCGCCACGCTTTATAGCGGTTAAT			
		pgi2F	CTGCTGGCGCTGATCGGCAT			
		pgi2R	TTATAGCGGTTAATCAGGCCGT			
*phoE*	Phosphoporine E	phoE604.1	ACCTACCGCAACACCGACTTCTTCGG	9	420	50
		phoE604.2	TGATCAGAACTGGTAGGTGAT			
*infB*	Translation initiation factor 2	infB1F	CTCGCTGCTGGACTATATTCG	6	318	50
		infB1R	CGCTTTCAGCTCAAGAACTTC			
		infB2F	ACTAAGGTTGCCTCCGGCGAAGC			
*tonB*	Periplasmic energy transducer	tonB1F	CTTTATACCTCGGTACATCAGGTT	17	414	50
		tonB2R	ATTCGCCGGCTGRGCRGAGAG			

a sequencing primers were the same as the PCR primers for *rpoB*, *gapA*, *mdh*, *phoE*, and *tonB*, while pgi2F/ 2R and infB2F/1R were the sequencing primers for *pgi* and *infB*, respectively.

**Table 2:** The correlation between different antimicrobial resistance determinants.

Antimicrobial resistant *Klebsiella* isolates	Strain(s) containing antimicrobial resistance determinants			*p*-value
90	Both PMQR and ESBL	PMQR	ESBL	
	77	1	9	0.0001
	Both ESBL and Integron 1	ESBL	Integron 1	
	76	10	1	0.0003
	Both PMQR and Integron 1	PMQR	Integron 1	
	71	7	6	0.0001
Transconjugants from antimicrobial resistant *Klebsiella* isolates	Both PMQR and Integron 1	PMQR	Integron 1	
86	43	14	13	0.0045

ESBL, extended-spectrum beta-lactamase gene; PMQR, plasmid-mediated quinolone resistance gene; integrons 1, class 1 integron.
