# Supplementary material for: Data on the phylogenetic typing, integron gene cassette array analysis, multi-drug resistance analysis and correlation between antimicrobial resistance determinants in Klebsiella strains
Source: Data Brief. 2016 Aug 3;8:1289–94. doi: 10.1016/j.dib.2016.07.016 (PMC4990640; doi:10.1016/j.dib.2016.07.016)
Supplement: Supplementary file 1 — Supplementary material [file mmc1.doc]

**Conflict of Interest**

The authors declare there is no conflict of interest.
